# Best practice guidelines for management of spinal disorders in skeletal dysplasia

**DOI:** 10.1186/s13023-020-01415-7

**Published:** 2020-06-24

**Authors:** Klane K. White, Michael B. Bober, Tae-Joon Cho, Michael J. Goldberg, Julie Hoover-Fong, Melita Irving, Shawn E. Kamps, William G. Mackenzie, Cathleen Raggio, Samantha A. Spencer, Viviana Bompadre, Ravi Savarirayan, on behalf of the Skeletal Dysplasia Management Consortium

**Affiliations:** 1 Department of Orthopedics and Sports Medicine, Seattle Children’s Hospital, 4800 Sand Point Way, OA.9.120, Seattle, Washington 98105 USA; 2 Department of Orthopaedics and Sports Medicine, University of Washington, Seattle, WA USA; 3 Division of Orthogenetics, Nemours/ A.I. duPont Hospital for Children, Wilmington, DE USA; 4 Division of Pediatric Orthopaedics, Seoul National University Children’s Hospital, Seoul, South Korea; 5 McKusick-Nathans Department of Genetic Medicine, Johns Hopkins University, Baltimore, MD USA; 6 Department of Clinical Genetics Guy’s and St Thomas NHS, London, UK; 7 Department of Radiology, Seattle Children’s Hospital, Seattle, WA USA; 8 Department of Radiology, University of Washington, Seattle, WA USA; 9 Department of Orthopedic Surgery, Nemours/ A.I. duPont Hospital for Children, Wilmington, DE USA; 10 Department of Orthopedic Surgery, Hospital for Special Surgery, New York, NY USA; 11 Department of Orthopedic Surgery, Boston Children’s Hospital, Boston, MA USA; 12 Victorian Clinical Genetics Services, Murdoch Children’s Research Institute, University of Melbourne, Parkville, Victoria Australia

**Keywords:** Skeletal dysplasia, Spine, Scoliosis, Kyphosis, Cervical instability, Spinal cord compression, Myelopathy

## Abstract

**Background:**

Disorders of the spine present a common and difficult management concern in patients with skeletal dysplasia. Due to the rarity of these conditions however, the literature, largely consisting of small, single institution case series, is sparse in regard to well-designed studies to support clinical decision making in these situations.

**Methods:**

Using the Delphi method, an international, multi-disciplinary group of individuals, with significant experience in the care of patients with skeletal dysplasia, convened to develop multi-disciplinary, “best practice” guidelines in the care of spinal disorders in patients with skeletal dysplasia.

**Results:**

Starting with 33 statements, the group a developed a list of 31 “best practice” guidelines.

**Conclusions:**

The guidelines are presented and discussed to provide context for clinicians in their decision making in this often-challenging realm of care.

## Background

Skeletal dysplasia is a heterogeneous group of over 500 genetically-mediated disorders that affect the development, growth and maintenance of bone and cartilage, leading to disproportionate short stature and abnormalities of the extremities and spine [1]. The 2019 nosology of skeletal disorders comprises 461 different diseases that are classified into 42 groups based on their clinical, radiographic, and/or molecular phenotypes. The most common skeletal dysplasia is achondroplasia, which is part of the FGFR3 chondrodysplasia group. This group, among others. Commonly manifest with spinal involvement. Those disorders most associated with spinal disease include, but are not limited to, the type II collagen disorders, sulphation disorders, Filamin B disorders, TRPV4 group, pseudoachondroplasia, bent bone dysplasia group, chondrodysplasia punctata group, osteogenesis imperfecta and the lysosomal storage diseases. (Table 1).

**Table 1:** Skeletal dysplasia with significant spinal manifestations^a^

Group/Name of Disorder	Inheritance	Gene	OMIM Number	ORPHANET Code	Typical Spinal Manifestations^**b**^
Achondroplasia	AD	FGFR3	100,800	18,060	3,5
Hypochondroplasia	AD	FGFR3	146,000	146,000	5
Spondyloepiphyseal dysplasia congenita (SEDC)	AD, AR	COL2A1	183,900	604,864 616,583	1,3,4
Kniest dysplasia	AD	COL2A1	156,550	485	1,3,4
Diastrophic dysplasia (DTD)	AR	SLC26A2	222,600	628	3,4
Atelosteogenesis type 3 (AO3)	AD	FLNB	108,721	56,305	3,4
Larsen syndrome (dominant)	AD	FLNB	150,250	503	3,4
Metatropic dysplasia	AD	TRPV4	156,530	2635	1,2,3,4
Pseudoachondroplasia (PSACH)	AD	COMP	177,170	750	1,4
Campomelic dysplasia (CD)	AD	SOX9	114,290	140	3,4
CDP, X-linked dominant, Conradi–Hünermann type (CDPX2)	XL	EBP	302,960	35,173	3,4
Osteogenesis imperfecta, progressively deforming type (OI type 3)	AD	COL1A1 COL1A2	259,420	216,812	4
Mucopolysaccharidosis type 1H	AR	IDUA	607,014	579	2,3
Mucopolysaccharidosis type 4A	AR	GALNS	253,000	309,297	1,3,4
Mucopolysaccharidosis type 6	AR	ARSB	253,200	583	1,2,3

^a^adapted from Mortier et al. [1]

^b^1-cervical instability, 2-cervical stenosis, 3- cervical/thoracic/thoracolumbar kyphosis, 4-scoliosis, 5-lumbar stenosis

Pathology of the spinal column appears where there is physeal growth, including the skull base (foramen magnum), odontoid process, neuro-central synchondrosis and vertebral endplates. Growth disturbance in these areas of the spine results in foramen magnum stenosis, atlantoaxial instability, spinal stenosis, kyphosis and scoliosis.

Disorders of the spine are a significant cause of morbidity and mortality in skeletal dysplasia [2–4]. Spine disorders may present early in life and are often progressive and severe. Spinal cord compression, with resultant paralysis or death, is the most pressing concern. Prevention of these complications requires early detection, close monitoring, and at times, prompt treatment. Their presence is often missed due to misdiagnosis or a lack of knowledge of the natural history of individual disorders. Treatment does not always follow established norms for spinal disease. Because of these factors, this working group set out to develop multi-disciplinary “best practice” guidelines for care for this complicated and poorly understood group of patients, to optimize health outcomes.

## Methods

A RAND-UCLA modified Delphi method was used to create consensus-based guidelines for the treatment and management of spine disease in patients with skeletal dysplasia. This methodology consists of a systematic literature review, creation of a list of statements, multiple rounds vetting those statements, and a face-to-face meeting where these statements are rated anonymously by a group of experts [5, 6].

Literature searches were executed in July 2018 in Ovid Medline and Embase. The search targeted literature on skeletal dysplasia and spine, abnormalities, or surgical procedures. Results were limited to English, humans, and items published 1998 to current. Some of the search terms included skeletal dysplasia, osteochondrodysplasias, achondroplasia, spondylometaphyseal dysplasia, spinal diseases, spinal curvatures, cervical vertebrae, scoliosis and kyphosis. A total of 1141 articles were first obtained, after refining the search 110 articles and 72 case reports were included in the review. Panelists added articles that were not included in this review.

The primary author (KKW) created statements based on a literature review. These were evaluated by other senior authors (MJG, WGM, RS) who edited the list. The first electronic survey consisted of 33 statements and was distributed with a referenced literature search to a panel of international experts in October 2018 (Round 1) (Additional file 1). Respondents rated the statements using a 5-point Likert scale (Strongly Agree, Agree, Neutral, Disagree and Disagree Strongly). A month later, at a face-to-face meeting, the results of the survey and the literature review were presented. Structured discussion was focused on areas of disagreement with the opportunity to modify statements if desired. The panel then suggested edits and the revised statements were rated anonymously for a second time by electronic survey (Round 2).

Consensus of 80% is considered the standard for Delphi processes, and this percentage was agreed to a priori. Statements with ≥80% of agreement between two categories (strongly agree and agree; or strongly disagree or disagree) were interpreted as appropriate or inappropriate and included in the guidelines. The panel consisted of 11 international experts on skeletal dysplasia comprising: 6 pediatric orthopedic surgeons; 4 medical geneticists; and 1 pediatric radiologist. One of the pediatric orthopedic surgeons did not participate in the second round.

## Results

In round one, the panel agreed on 19 of 33 statements (Additional file 1). Statements not reaching 80% agreement are listed in Additional file 1. During the face-to face meeting, the list was modified into 32 statements and rated again (round two), where 31 statements reached ≥80% consensus (Table 2) and 1 statement did not (Table 3).

**Table 2:** Statements that reached 80% agreement in Round 2. Final Guidelines

	Strongly Agree	Agree	Neutral	Disagree	Strongly Disagree
1.Spinal disorders are common in skeletal dysplasia.	8 (80%)	2 (20%)	0	0	0
2.Spinal disorders can have an infantile onset (age 0–3 years) and are often progressive in nature.	7 (70%)	3 (30%)	0	0	0
3.Spinal cord compression and myelopathy are common manifestations of spinal disorders in skeletal dysplasia.	3 (30%)	7 (70%)			
4.Myelopathic findings on history and physical exam (e.g. poor balance, broad based gait, extremity weakness, upper motor neuron signs, urinary incontinence) should raise suspicion of spinal cord compression/injury in patients with skeletal dysplasia.	10 (100%)	0	0	0	0
5.Clinical evidence of myelopathy requires urgent evaluation and management.	9 (90%)	1 (10%)	0	0	0
6.In patients with skeletal dysplasia and “spine-at-risk”* findings, neuromonitoring should be considered for all surgical procedures to minimize the risk of spinal cord injury.	3 (30%)	7 (70%)	0	0	0
7.Skeletal dysplasia should be considered in individuals with radiographic findings of vertebral anomalies such as platyspondyly and/or anterior vertebral body beaking.	6 (60%)	4 (40%)	0	0	0
8.Achondroplasia or hypochondroplasia are likely diagnoses if there narrowing of the interpedicular distance in the lumbar spine (from L1 to L5) on AP radiographs.	6 (60%)	4 (40%)	0	0	0
9.Flexion/extension plain radiographs of the cervical spine should be considered for all patients with known risk of C1-C2 instability or unclassified skeletal dysplasia.	9 (90%)	1 (10%)	0	0	0
10.Vertebral artery and upper cervical anatomy is variable in skeletal dysplasia; therefore advanced imaging is recommended prior to upper cervical spinal surgery.	6 (60%)	4 (40%)	0	0	0
11.Flexion-extension CT scan or MRI can be very useful adjuncts in evaluating cervical instability in patients with skeletal dysplasia.	8 (80%)	2 (20%)	0	0	0
12.Cervical instability or evidence of significant spinal cord compression on imaging associated with myelopathic changes on physical exam should be considered for surgical management.	10 (100%)	0	0	0	0
13.Prophylactic C1-C2 fusion for an individual at risk for cervical instability is not indicated without evidence of spinal cord compression or myelopathic changes.	7 (70%)	3 (30%)	0	0	0
14.There are several effective techniques for stabilization of the cervical spine in patients with skeletal dysplasia. Treating surgeons should be prepared for unusual anatomy in this patient population.	9 (90%)	1 (10%)	0	0	0
15.Stenosis may occur at any level in the cervical spine in skeletal dysplasia.	9 (90%)	1 (10%)	0	0	0
16.Cervical kyphosis can be seen in skeletal dysplasia. Repeated evaluation is indicated as progression may occur and lead to spinal cord injury if untreated.	9 (90%)	1 (10%)	0	0	0
17.Upper thoracic kyphosis occurs in skeletal dysplasia and can be associated with spinal cord injury during procedures requiring anesthesia.	6 (60%)	4 (40%)	0	0	0
18.Thoracolumbar kyphosis in infants with achondroplasia improves in most cases without bracing or surgery, but prolonged unsupported sitting is discouraged.	8 (80%)	2 (20%)	0	0	0
19.Thoracolumbar kyphosis can be seen in skeletal dysplasia. Repeated evaluation is indicated as progression may occur and lead to neurologic symptoms or back pain if untreated.	9 (90%)	1 (10%)	0	0	0
20.Surgical stabilization of thoracolumbar kyphosis in skeletal dysplasia is appropriate for deformities that are progressive, result in neurologic compromise, or associated with back pain not responsive to non-operative interventions.	7 (70%)	3 (30%)	0	0	0
21.Instrumented fusion with or without decompression for thoracolumbar kyphosis in skeletal dysplasia is most successful when sagittal alignment and balance are achieved.	6 (60%)	4 (40%)	0	0	0
22.Respiratory function should be monitored in patients with thoracic spinal deformity.	3 (30%)	7 (70%)	0	0	0
23.Brace or cast treatment in skeletal dysplasia is appropriate in young patients with progressive, flexible scoliosis.	4 (40%)	6 (60%)	0	0	0
24.Early-onset scoliosis occurs in skeletal dysplasia and can be managed with surgical techniques that preserve spine growth.	6 (60%)	4 (40%)	0	0	0
25.Surgical management of scoliosis and kyphosis in skeletal dysplasia is associated with a higher complication rate compared to the general population.	5 (50%)	5 (50%)	0	0	0
26.Advanced imaging is strongly recommended prior to surgical instrumentation of the spine in skeletal dysplasia.	8 (80%)	2 (20%)	0	0	0
27.In achondroplasia, symptomatic spinal stenosis can present in the upper and lower extremities. Symptoms and signs include decreased strength or mobility, neurogenic claudication, back and leg pain, and/or upper and lower motor neuron findings.	8 (80%)	2 (20%)	0	0	0
28.Progressive symptoms and signs of spinal stenosis causing reduced physical function in achondroplasia should be treated surgically by decompression when appropriate non-operative measures are ineffective.	3 (30%)	7 (70%)	0	0	0
29.Surgical decompression should be accompanied by instrumented fusion in skeletally immature patients with achondroplasia and progressive symptomatic spinal stenosis.	4 (40%)	6 (60%)	0	0	0
30.In hypochondroplasia, symptomatic spinal stenosis can occur and should be monitored.	6 (60%)	4 (40%)	0	0	0
31.Increased lumbar lordosis can be associated with hip flexion contractures. Realignment of the hip deformity can improve sagittal alignment of the spine.	7 (70%)	2 (20%)	1 (10%)	0	0

**Table 3:** Statement that did not reach 80% agreement in Round 2

	Strongly Agree	Agree	Neutral	Disagree	Strongly Disagree
Skeletal dysplasia patients with known spinal disease require routine evaluation and surveillance with MRI of the entire spine.	2 (20%)	5 (50%)	2 (20%)	1 (10%)	0

The Delphi process substantially increased consensus for modified statements and a list of 31 “best practice” guidelines were generated (Table 2). After review of results, all participants agreed to support its publication.

## Discussion

### General considerations

Spine disorders in skeletal dysplasia are caused by abnormalities to development, growth or maintenance of the bony elements of the spine, and are present in the majority of conditions included in the latest International Skeletal Dysplasia Society nosology [1]. Spinal disorders include kyphosis, scoliosis, central stenosis, vertebral instability and hyperlordosis, often resulting in neurologic decompensation. Their presentation can be extremely variable within and between different types of skeletal dysplasia, for example in disorders of type II collagen [7].

It is important to clinically examine the spine in all cases where a skeletal dysplasia is suspected or confirmed and perform appropriate or indicated radiographic evaluation as described subsequently. Spine disorders associated with skeletal dysplasia can present at a very young age. As many of these spinal disorders can be progressive, ongoing clinical and radiographic surveillance is encouraged.

Due to abnormal shape, size, and growth of the bony elements that comprise the spinal column in any skeletal dysplasia with spinal disease, there is increased risk of spinal cord compression. This may remain asymptomatic, or present as clinical signs of myelopathy, comprising poor balance, unsteady gait, extremity weakness, upper motor neuron signs, and urinary/bowel incontinence. Therefore, in any individual with skeletal dysplasia and spinal manifestations, careful neurological examination should be regularly performed to assess for the presence of myelopathy. Recognition of the symptoms or signs of myelopathy in an individual with skeletal dysplasia requires further evaluation, as catastrophic injury from intrinsic canal structure and minor trauma can occur [8]. Once the level of myelopathy is identified, appropriate steps to manage the spinal cord and canal structure can be determined.

In achondroplasia, the most common form of disproportionate short stature, symptomatic spinal stenosis can occur at any level throughout the spine [9] and is not necessarily isolated to the lumbar region [10]. Therefore, symptom inquiry and physical examination should reflect this. In the lumbar spine, stenosis results in lower motor symptoms of neurogenic claudication, evident as tingling, pain or cramping in the back, buttocks and legs (typically relieved by squatting or leaning forward), and lower limb weakness [4]. Thoracic spine stenosis can manifest via upper motor neuron symptoms such as urinary incontinence, bowel dysfunction and gait disturbance, with lower limb spasticity and hyperreflexia, clonus and the Babinski sign [4]. Abnormal upper limb neurology is uncommon, but does occur [9, 11]. The resulting cervical myelopathy is may be associated with pain and stiffness in the neck, tingling, weakness or numbness in the hands, loss of fine motor dexterity, poor balance and vertigo, in addition to those signs and symptoms resulting from thoracic level disease.

In previous recommendations provided by this work group, the concept of “spine-at-risk” was introduced with regard to risk of spinal cord injury related to prolonged anesthesia [12]. There are now several case reports of spinal cord infarct after with prolonged neurosurgical or orthopedic procedures, in areas separate from the primary site of operation [13–16]. “Spine-at-risk” findings in the pre-operative period include: foramen magnum stenosis, atlantoaxial instability, cervical stenosis, cervical kyphosis, cervicothoracic kyphosis, thoracic level stenosis, cord level thoracolumbar kyphosis, syrinx, and cord signal abnormalities on MRI. The area of greatest risk appears to be at the upper thoracic level. For the purposes of this discussion, the workgroup defined prolonged anesthesia as a duration exceeding 1 h. For practical reasons, neuromonitoring is not recommended for procedures lasting less than 1 h. The opinion of the group is that the risk of permanent neurologic deficit after shorter procedures (e.g. placement of ear tubes, endoscopy, etc.) is small, and the set-up of neuromonitoring before such cases is therefore impractical.

### Imaging

The vertebrae in skeletal dysplasia are characteristically dysplastic, which clinically can result in short trunk dwarfism and associated scoliosis or kyphosis. Many types of skeletal dysplasia also have hypoplasia of the odontoid process with associated craniocervical instability [17]. Some skeletal dysplasia are suspected antenatally on ultrasound findings, which can be further delineated by prenatal MRI [7].

Common vertebral shape anomalies in the context of skeletal dysplasia include platyspondyly, anterior vertebral body beaking, coronal clefting, narrowing of the interpediculate distance in lumbar vertebrae, and posterior scalloping of the vertebral body. These findings should be interpreted in the context of other radiographic and clinical findings when generating a differential diagnosis.

Platyspondyly may be seen as a solitary vertebral finding, or in conjunction with other skeletal features. Some of the more common types of dysplasia that demonstrate platyspondyly, include pseudoachondroplasia, metatropic dysplasia, and type II collagen disorders such as Kniest dysplasia (with coronal clefting) [18] and spondyloepiphyseal dysplasia congenita (SEDC). Other forms of skeletal dysplasia with platyspondyly include the spondylometaphyseal dysplasia and lethal dysplasia such as thanatophoric dysplasia and short-rib polydactyly [19].

Anterior vertebral body beaking is typical of pseudoachondroplasia and the mucopolysaccharidoses (MPS). In Hurler syndrome (MPS 1H) beaking often occurs at the inferior aspect of the anterior vertebra, while Morquio syndrome (MPS IV) and pseudoachodroplasia are often associated with beaking of the middle portion of the anterior vertebra [20]. When any beaking is present, MPS should be considered, and screening of leukocyte enzyme activity and urinary glycosaminoglycans should be obtained to rule out these treatable disorders.

The most common skeletal dysplasia, achondroplasia, has characteristic narrowing (or failure of normal widening) of the interpedicular distance in the caudal portion of the lumbar spine relative to the cephalad portion on antero-posterior (AP) radiographs. Due to abnormal endochondral ossification, the axial skeleton also demonstrates shortened pedicles and narrowing of the bony spinal canal with posterior vertebral body scalloping (best seen on lateral radiographs), including narrowing at the level of the foramen magnum [2].

Less commonly interpedicular narrowing may be found in other entities with *FGFR3* mutations, such as hypochondroplasia and the lethal thanatophoric dysplasia. Diastrophic dysplasia is also characterized by interpedicular narrowing of the lumbar spine but does not share other radiographic features of skeletal dysplasia with *FGFR3* mutations, instead being characterized by severe epiphyseal dysplasia and marked progressive kyphoscoliosis.

Flexion-extension cervical spine films are useful in clinical practice to assess for instability of the occipital-cervical junction. Anyone with a short stature skeletal dysplasia that includes hypoplasia of the odontoid, axis or other cervical vertebrae is at risk of instability that could predispose to spinal cord injury. Examples of skeletal dysplasia with known risk of cervical instability include SEDC, pseudoachondroplasia, diastrophic dysplasia, metatropic dysplasia and Morquio syndrome [21]. As soon as a young child can cooperate to perform flexion-extension radiographs of the cervical spine (typically age 2–3 years), imaging should be done to define cervical spine stability. Until then, infants and young children with these diagnoses should be managed with close clinical surveillance until cervical instability can be excluded. Full initial assessment of all patients with a suspected skeletal dysplasia, including older children and/or adults who do not have a known dysplasia diagnosis, as they may be at risk of unrecognized cervical stenosis or instability.

Flexion-extension plain films of the cervical spine in children with skeletal dysplasia can be difficult to interpret due to incomplete vertebral ossification, overlapping structures, and abnormal anatomy such as hypoplastic dens or os odontoideum [22, 23]. Plain films also lack the ability to evaluate the underlying spinal cord in the setting of cervical instability. Flexion-extension MRI has been shown to be a safe method of evaluating cervical instability in children without skeletal dysplasia [24]. Flexion-extension MRI of the cervical spine under sedation/anesthesia is helpful in skeletal dysplasia patients with abnormal neurological examination in detecting suspected instability, stenosis, or inconclusive findings on flexion-extension radiographs, and in assessing cord compression and presence of unsuspected os odontoideum [22, 25]. While dynamic CT imaging cannot evaluate the spinal cord as well as MRI, visualization of bony anatomy and assessment of translation during flexion and extension can be improved as compared to plain films alone.

MRI of the entire spine is an important diagnostic method in skeletal dysplasia patients with neurologic signs or symptoms. However, the group did not agree on its use in routine surveillance for patients with skeletal dysplasia known to be associated with spinal disease. No clear literature exists on the topic, but expert opinion recommendations for MPS IVA (Morquio syndrome) support surveillance MRI at least every 3 years for patients on enzyme replacement therapy [23]. As such, some members thought it appropriate to recommend routine surveillance imaging. Because of the risk of equivocal imaging findings, often in the absence of clear clinical detriment, more conservative panelists believed that it is enough to obtain whole spine MRI only when neurologic signs or symptoms ensue to clarify the role for intervention.

As alluded to previously, there are multiple reports of upper thoracic spinal cord infarct in patients with skeletal dysplasia after prolonged anesthesia [13–16]. Four out of five of these patients had a form of MPS, the other, SEDC. The kyphotic deformity can often be subtle, as can the cord effacement over a prominent intervertebral disc or vertebral body as imaged on MRI. These findings should be evaluated for and be considered concerning for risk of neurologic injury during lower extremity surgery.

Variations in the course of the vertebral artery have been described in skeletal dysplasia. Hypoplastic posterior elements, delayed ossification, small pedicles, odontoid hypoplasia, os odontoideum and upper cervical instability can all result in atypical anatomy. This may modify the surgical approaches and instrumentation [26]. Three-dimensional imaging and angiography with CT and/or MRI are essential to accurately assess the anatomy and plan surgery. Intraoperative portable CT machines allow 3-dimensional assessment of anatomy and guided instrumentation during surgery. Intraoperative MRI can also be utilized for non-instrumented cases such as foramen magnum decompression.

### Medical management

The wide variety of spinal manifestations seen in skeletal dysplasia patients may be seen at birth, while in others they evolve as the skeleton grows. At present, no medications have demonstrated reduction in spinal disease. Thus, serial physical examinations and radiographic studies are essential from birth to determine the age of onset, rate of progression and overall severity of spinal problems in skeletal dysplasia patients. Non-surgical management may be appropriate for some spinal complications for a while, but close observation is needed to assess if and when surgery is indicated to optimize function and minimize pain. In the event of progressive worsening, active management is essential to prevent permanent neurological sequelae. In the first instance, anticipatory non-operative measures should be implemented concurrent with ongoing surveillance. These measures would include physical therapy, weight reduction and the use of appropriate non-steroidal inflammatory analgesia. If in spite of these interventions, close monitoring indicates further progression, surgery should be undertaken [4, 9, 10, 27]. Since the neurological complications of bowel dysfunction, urinary incontinence and spasticity may not recover following surgery, the severity should not be permitted to progress to the point that function cannot be restored.

Symptoms and signs of spinal stenosis require close and regular monitoring. Multiple levels of spinal cord compression can occur in various skeletal dysplasia. Rhizomelic chondrodysplasia punctata, MPS and metatropic dysplasia can also have multi-level stenosis at a young age. *FGFR3* disorders can have severe congenital stenosis that can manifest at any age resulting in delayed motor development and myelomalacia. Progressive deformity, such as with cervical kyphosis in diastrophic dysplasia, campomelic dysplasia, Kniest dysplasia, atelosteogenesis and Larsen spectrum disorders, or thoracolumbar kyphosis in achondroplasia, is often associated with stenosis as well. Soft tissue as well as bony impingement can occur. Management is according to neurologic symptoms and severity of cord compression and/or myelopathy.

Progressive cervical kyphosis may cause severe cord compression and quadriplegia. Careful surveillance is required. The natural history of cervical kyphosis in diastrophic dysplasia is resolution by age 5 in 75% [28]. In the absence of ovoid apical vertebra and curve magnitude < 60 degrees, progression is unlikely and serial evaluation is recommended. Thoracolumbar kyphosis (TLK) is universally observed in infants with achondroplasia. TLK is thought to be a result of developmental motor delay and hypotonia and is evident from birth. It is believed that the majority improve: 15% by walking age and 60% by 1 year after walking [29, 30]. Other studies have suggested that 30% of TLK persist with one-third of those (10% overall) progressing to severe deformity. Borkhuu et al. reported that progressive curves were significantly associated with developmental motor delay (walkers after 24 months compared to 18 months), apical vertebral wedging and apical vertebral translation. Bracing with thoraco-lumbar-sacral orthoses (TLSOs) has been used in this population and is commonly employed in persistent deformity [31]. Unsupported sitting is discouraged to eliminate anterior vertebral body compression [31]. To minimize the likelihood of vertebral body wedging, anticipatory guidance regarding posture in the care of these infants is recommended in early infancy [32].

A fraction of patients with achondroplasia will develop symptomatic, fixed deformities requiring surgery. A similar deformity is present in over 90% of children with MPS I-H (Hurler syndrome), and can be seen in many other forms of skeletal dysplasia [29, 30, 33]. In MPS I-H progression is more likely in kyphoses exceeding 45°, but is not universal [34]. Persistence and progression of kyphosis beyond childhood can lead to neurologic compromise and paraspinal muscular back pain [35]. Clinical surveillance with periodic plain radiographic imaging is encouraged to monitor for progression. The group was not prescriptive in the appropriate intervals for evaluation, deferring to the treating clinician to decide, depending on individual patient presentations and needs.

Persistence of kyphosis may lead to myelopathy, paraparesis or back pain [35]. With that said, the majority of thoracolumbar kyphosis occur below the level of the conus medullaris, and the risk of spinal cord compression or other neurologic compromise in young children is low. For older children and adolescents, the presence of thoracolumbar kyphosis can exacerbate the symptoms of neurogenic claudication in achondroplasia [30, 36].

Spinal deformity may negatively impact the size of the thorax and pulmonary function. Many of the features of skeletal dysplasia are progressive in nature and therefore pulmonary compromise may evolve and worsen over time. If pulmonary function deteriorates, medical therapies (e.g. non-invasive positive pressure ventilation) or surgical correction of kyphosis or scoliosis may be indicated. In observance of the principles of early onset scoliosis management, spinal growth preservation is essential while preventing progression of severe spinal deformity [37]. In the experience of the workgroup, young patients with skeletal dysplasia and flexible spinal deformity respond well to bracing and serial casting.

It should be noted that there are now several promising emerging drug therapies for individuals with skeletal dysplasia that might alter their natural history, surgical management, and long-term outcomes with regard to spinal disease, however to date none has shown any significant benefit with regard to spinal disease [38, 39].

### Surgical management

Progressive myelopathy occurs over time in the presence of persisting instability and cord compression. To prevent permanent injury, surgical stabilization should be performed when it is suspected. If cervical instability reduces without cord compression, then instrumented fusion without decompression can be considered. If cord compression is observed after reduction, decompression should be performed before instrumented fusion.

Although upper cervical instability is common in skeletal dysplasia, progression and severity is variable. Surgery is challenging and complex in these patients. Therefore, patients at risk of instability should be followed with serial imaging and a careful neurological assessment. Surgical intervention for cervical instability should be reserved for demonstrable pathology rather than performed prophylactically. The risk of waiting for symptoms too long is permanent myelopathy; therefore, vigilant surveillance is suggested.

In pediatric cervical spine surgery, instrumentation promotes higher fusion rates [40]. Historical techniques without instrumentation and bone grafting with halo immobilization are less effective but are sometimes required due to the hypoplastic bony elements and small size of the patients. Current cervical instrumentation can be used in pediatric patients. Occipital plates, multi-axial lateral mass, intralaminar, transarticular and pedicle screws allow various construct options.

The opinion of the group is that surgical intervention for thoracolumbar kyphosis should be reserved for deformities that are progressive or symptomatic (i.e. neurological compromise or secondary muscular back pain). Indications in the literature for surgical stabilization of these deformities have varied. Early recommendations for fusion include persistence of triangular-shaped apical vertebrae at walking age or thoracolumbar kyphosis greater than 30°, “delaying” correction to age 4 or 5 years. Lonstein advocated for fusion of angular or progressive kyphosis or for kyphosis present at the time of decompression for spinal stenosis [41–43]. Ain et al. [36] recommended surgery when thoracolumbar kyphosis exceeded 60° with more than 10° of progression per year. For MPS I-H surgical stabilization of thoracolumbar kyphosis has been recommended for extreme deformity progression (> 70°) or neurologic compromise [44, 45]. Others have utilized less conservative criteria, such as deformity progression of > 10 degrees over a 12-month period with disruption of sagittal balance despite brace treatment [46]. Given the inherent risks of surgery such as junctional deformity, infection and neurologic injury, balanced with the limited detriment to observation and the benefits of continued growth, we feel relatively conservative recommendations are appropriate. For this group surgery may be delayed until deformities are symptomatic (i.e. pain, neurologic compromise) or when there is continued progression beyond 60°-70°. These procedures can be often be delayed well into late childhood, if not adolescence, regardless of skeletal dysplasia, making the anatomy much more approachable and allowing continued growth of the spine.

There is a lack of consensus in the literature about the need for both anterior and posterior approaches in the treatment of kyphotic deformities, particularly with regard to those in skeletal dysplasia. Historically, recommendations for surgical treatment of thoracolumbar kyphosis in achondroplasia have suggested minimizing correction and avoiding posterior instrumentation because of the high risk of neurologic injury [42, 43]. With the popularization of pedicle screws, however, these surgeries have become safer, even in young children [47]. Posterior approaches alone however, without shortening of the posterior column, risk lengthening the spinal cord and consequent neurological injury in more severe deformities [48]. Additionally, isolated short segment posterior fusion without appropriate deformity correction increases the risk of junctional deformity and the need for revision surgery.

Full deformity correction through vertebral column resection via combined anterior and posterior approach, or through an all-posterior approach, both allow for correction of most deformities and avoid lengthening of the spinal cord [49, 50]. Ain and Shirley reported good results with posterior only instrumentation and fusion in younger children with achondroplasia [51]. In MPS I-H, three-column correction has been recommended by either a combined anterior-posterior procedure or by pedicle subtraction osteotomy [52, 53]. More recently a posterior only approach has been advocated by Bekmez et al. [43, 46] Careful scrutiny of this paper does not necessarily support these conclusions, however. Of the six patients included, two were revisions for failed posterior only procedures and two others required subsequent revision for junctional deformity. Consequently, this group strongly advocates caution in considering a posterior only approach that does not address the anterior column of the spine.

Considering the literature, and the collective experience of the work group, it was agreed that successful treatment relies less on the approach, and more on the resultant sagittal alignment at the site of correction, and overall spinal balance. For less severe and more flexible deformities, such as those found in young children, isolated posterior fusion and instrumentation can be successful. However, we feel that surgical treatment in this group should be an uncommon occurrence. Older children, adolescents, and adults with symptomatic deformities may require more aggressive surgical correction. Consequently, the approach to achieve appropriate sagittal alignment and balance relies less any prescribed recommendation, and more on the experience of the surgeon and the perceived risk of surgery in that surgeons’ hands in attaining appropriate correction. Management of spinal deformity in skeletal dysplasia has higher complication rates, such as 30% incidence of dural tear, 20% neurological injury, and undefined but high rates of recurrent symptomatic stenosis in achondroplasia. Instrumentation failure and junctional kyphosis can be seen in instrumented fusions as previously discussed.

Growth-friendly spinal instrumentation has been shown to be effective in skeletally immature patients with skeletal dysplasia [54]. A higher complication and revision rate are reported in skeletal dysplasia patients with severe spine deformity, however in general, control of deformity and trunk growth can be achieved [55]. In skeletally immature patients with achondroplasia and symptomatic spinal stenosis, decompression without fusion yields progressive kyphosis [21]. In those with stenosis and pre-existing kyphosis, decompression alone will cause rapid progression of deformity. Therefore, any surgical treatment of symptomatic stenosis in these patients should include instrumented spinal fusion with appropriate correction of sagittal plane deformity. Spinal stenosis secondary to inadequate widening of the spinal canal, whilst typical in achondroplasia, can also occur in hypochondroplasia, albeit rarely [56, 57]. As such, symptomatic inquiry in individuals with hypochondroplasia should encompass the possibility of spinal stenosis, although routine monitoring for this is not necessary.

Hip contracture results in a forward tilt of the pelvis and compensatory lumbar lordosis to maintain sagittal plane balance. Bayhan et al. demonstrated correction of lumbar lordosis in their series of SEDC patients undergoing valgus-extension proximal femoral osteotomies. In this study hip flexion contractures improved from preoperative average 16 degrees to 3 degrees, lumbar lordosis improved from preoperative average 81 degrees 70 degrees, sacral slope from 55 degrees to 48 degrees, pelvic tilt from − 29 degrees to − 6 degrees, and pelvic incidence from 26 degrees to 42 degrees [58]. This benefit has been limited to type II collagen disorders and not demonstrated for others, such as achondroplasia.

## Conclusion

Given the rarity of these disorders, it is the aim of this working group to provide clinicians with multi-disciplinary guidelines for diagnosis, evaluation and treatment of patients with skeletal dysplasia with spinal pathology. The use of expert opinion at this time serves as a stopgap to fill the dearth of relevant evidence in the literature. These guidelines also provide opportunities to identify gaps in knowledge that will serve as a springboard for further investigation.

## Supplementary information

**Additional file 1.** Statements that did and did not reached 80% agreement in Round 1.

## Data Availability

All data generated or analyzed during this study are included in this published article.
