# Supplementary material for: Best practice guidelines for management of spinal disorders in skeletal dysplasia
Source: Orphanet J Rare Dis. 2020 Jun 24;15:161. doi: 10.1186/s13023-020-01415-7 (PMC7313125; doi:10.1186/s13023-020-01415-7)
Supplement: Supplementary file 1 — Additional file 1. Statements that did and did not reached 80% agreement in Round 1. [file 13023_2020_1415_MOESM1_ESM.docx]

|  | Strongly Agree | Agree | Neutral | Disagree | Strongly Disagree |
| --- | --- | --- | --- | --- | --- |
| 1.Spinal disorders are common is common in skeletal dysplasia. | 9 (82%) | 2(18%) | 0 | 0 | 0 |
| 2.Spinal disorders often presents early and is for some forms of skeletal dysplasia progressive in nature. | 9 (82%) | 2 (18%) | 0 | 0 | 0 |
| 3.Spinal cord compression and myelopathy are common manifestations of spinal disorders in skeletal dysplasia. | 4 (36%) | 6 (55%) | 0 | 1 (9%) | 0 |
| 4.Myelopathic findings on physical exam (poor balance, broad based gait, extremity weakness, upper motor neuron signs, urinary incontinence) should raise suspicion of spinal cord compression/injury in patients with skeletal dysplasia. | 10 (91%) | 1 (9%) | 0 | 0 | 0 |
| 5.Myelopathic findings on physical exam (poor balance, broad based gait, extremity weakness, upper motor neuron signs, urinary incontinence) should generally direct a more aggressive approach to treatment of spinal disease in patients with skeletal dysplasia. | 7 (64%) | 3 (27%) | 0 | 1 (9%) | 0 |
| 6.Spinal cord monitoring should be considered for prolonged (> 1 hour) anesthesia in children with spine deformity or instability to avoid spinal cord infarct. | 6 (55%) | 3 (27%) | 1 (9%) | 1 (9%) | 0 |
| 7.Platyspondyly and/or vertebral body hypoplasia are typical radiographic findings in skeletal dysplasia. | 3 (27%) | 7 (64%) | 1 (9%) | 0 | 0 |
| 8.Progressive narrowing of the interpediculate distance in the lumbar spine is highly suggestive of a diagnosis of achondroplasia or hypochondroplasia. | 4 (36%) | 6 (55%) | 0 | 1 (9%) | 0 |
| 9.Flexion-extension CT scan or MRI can be very useful adjuncts in evaluating cervical instability in patients with skeletal dysplasia. | 4 (36%) | 7 (64%) | 0 | 0 | 0 |
| 10.Cervical instability associated with myelopathic changes on physical exam, or evidence of significant spinal cord compression on imaging, should be considered for surgical stabilization. | 8 (73%) | 2 (18%) | 1 (9%) | 0 | 0 |
| 11.There are many effective techniques for stabilization of the cervical spine in patients with skeletal dysplasia. Treating surgeons should be prepared for unusual anatomy in this patient population. | 7 (64%) | 4 (36%) | 0 | 0 | 0 |
| 12.Cervical stenosis may occur in the subaxial spine in patients with skeletal dysplasia | 5 (46%) | 5 (45%) | 1 (9%) | 0 | 0 |
| 13.Thoracolumbar kyphosis in infants with achondroplasia resolves without treatment (including bracing) in the vast majority of cases. | 6 (55%) | 4 (36%) | 1 (9%) | 0 | 0 |
| 14.Surgical stabilization of thoracolumbar kyphosis in patients with skeletal dysplasia is appropriate in deformities that are progressive, result in neurologic compromise, or are associated with low back pain not responsive to non-operative interventions. | 3 (37%) | 8 (73%) | 0 | 0 | 0 |
| 15.Brace or cast treatment of scoliosis in patients with skeletal dysplasia is appropriate in young patients with progressive, flexible scoliosis. | 2 (18%) | 7 (64%) | 2 (18%) | 0 | 0 |
| 16.Surgical management of scoliosis and kyphosis in patients with skeletal dysplasia is associated with a higher complication rate than the general population. | 3 (27%) | 7 (64%) | 1 (9%) | 0 | 0 |
| 17. The spinal canal must be assessed preoperatively to avoid problems with instrumentation in the canal. | 5 (46%) | 4 (36%) | 1 (9%) | 1 (9%) | 0 |
| 18.In achondroplasia spinal claudication can present with decreased mobility, pain, and neurological lower limb changes. | 7 (64%) | 4 (36%) | 0 | 0 | 0 |
| 19.In hypochondroplasia, spinal stenosis can occur and should be monitored for clinically. | 3 (27%) | 5 (55%) | 1 (9%) | 0 | 1 (9%) |

Table 1. Statements that reached 80% agreement in Round 1.

Table 2. Statements that did not reach 80% agreement in round 1.

|  | Strongly Agree | Agree | Neutral | Disagree | Strongly Disagree |
| --- | --- | --- | --- | --- | --- |
| 1.All patients with skeletal dysplasia should be screened for cervical abnormalities with lateral view flexion/extension plain radiographs of the cervical spine prior to age 2 years. | 2 (18%) | 3 (27%) | 3 (27%) | 2 (18%) | 1 (10%) |
| 2.Skeletal dysplasia patients with known spinal disease require routine evaluation and surveillance with MRI of the entire spine. | 2 (18%) | 3 (27%) | 2 (18%) | 4 (37%) | 0 |
| 3.CT angiogram to evaluate for surgical anatomy and variable course of the vertebral artery is recommended prior to any cervical spine surgery. | 2 (19%) | 3 (27%) | 3 (27%) | 3 (27%) | 0 |
| 4.Prophylactic fusion for cervical instability, in the absence of signs or symptoms, is not warranted in Morquio syndrome (MPS IVA). | 1 (9%) | 5 (46%) | 1 (9%) | 4 (36%) | 0 |
| 5.Cervical kyphosis is most common in diastrophic dysplasia and Larsen syndrome. Cervical kyphosis in diastrophic dysplasia often improves with age and can typically be observed. In other skeletal dysplasias, cervical kyphosis is generally progressive and can result in spinal cord injury if left untreated. | 1 (9%) | 7 (64%) | 2 (18%) | 1 (9%) | 0 |
| 6.Cervicothoracic kyphosis is generally underappreciated in patients with skeletal dysplasia but is associated with spinal cord injury with procedures remote to this deformity (e.g. cervical spine, thoracolumbar spine and lower extremities). | 3 (27%) | 5 (46%) | 3 (27%) | 0 | 0 |
| 7.Thoracolumbar kyphosis in patients with skeletal dysplasia may be progressive in deformities exceeding 45 degrees. | 0 | 8 (73%) | 3 (27%) | 0 | 0 |
| 8.Thoracolumbar kyphosis in patients with skeletal dysplasia that occurs caudal to the conus medullaris can be observed avoiding early posterior fusion and associated growth restrictions | 1 (9%) | 4 (36%) | 5 (46%) | 1 (9%) | 0 |
| 9.Surgical stabilization of thoracolumbar kyphosis in patients with skeletal dysplasia is most successful when addressing all three columns of the spine. | 2 (18%) | 5 (46%) | 4 (36%) | 0 | 0 |
| 10.Scoliosis is common in patients with skeletal dysplasia and may exacerbate preexisting restrictive lung disease. | 3 (27%) | 4 (36%) | 0 | 4 (36%) | 0 |
| 11.Early Onset Scoliosis (EOS) is common in patients with skeletal dysplasia and is appropriately responsive to growth friendly techniques. | 1 (9%) | 4 (36%) | 4 (36%) | 2 (18%) | 0 |
| 12.Symptomatic spinal stenosis in achondroplasia should be treated surgically by decompression. | 2 (18%) | 5 (46%) | 3 (27%) | 1 (9%) | 0 |
| 13.Symptomatic spinal stenosis in achondroplasia in skeletally immature patients should be treated surgically by decompression and fusion. | 1 (9%) | 5 (46%) | 2 (18%) | 3 (27%) | 0 |
| 14.Lumbar lordosis associated with coxa vara (i.e. type II collagen disorders) is best addressed by treating hip deformity. | 2 (18%) | 6 (55%) | 2 (18%) | 1 (9%) | 0 |
